# Antipsychotic drug prescription in postgraduate psychiatry training programs in India: Time to reflect

**DOI:** 10.4103/0019-5545.37329

**Published:** 2007

**Authors:** Sunny T. Varghese

**Affiliations:** Department of Psychiatry, Amrita Institute of Medical Sciences and Research Centre, Cochin - 682 026, Kerala, India

The current postgraduate psychiatric training programs all over India are psychopharmacology oriented and are essentially based on the medical model of illness. An interesting feature of this change in psychiatric teaching programs is the comfort level of new graduates with typical antipsychotic medications. Most of the institutes and regional medical colleges in India currently emphasize the use of atypical antipsychotics for treatment of schizophrenia as suggested either by the American Psychiatric Association Guidelines or the National Institute of Clinical Excellence or the guidelines suggested by the Indian Psychiatric Society.

Among the recently passed postgraduates in Psychiatry, those who are equally comfortable with the use of typical and atypical antipsychotics are very few. The residents passing from leading institutes in India would have difficulty in prescribing Chlorpromazine or pimozide or trifluoperazine or even haloperidol. With new studies questioning the infallibility of the atypical antipsychotics, it should not come as a surprise if typical antipsychotics are again brought to the forefront in the management of schizophrenia.[1,2]

It may not be long when we would have created a generation of psychiatrists in India who would be poorly trained in psychotherapeutic techniques, as well as prescription of a group of medications which are essential drugs in our armamentarium against schizophrenia. All the Psychiatry program directors in India should be aware of this lacuna in our Psychiatry training, and adequate steps should be taken to rectify this.
